# Ultrastructural Changes in Hepatocytes and Chemopreventive Effects of Short-Term Administration of *Curcuma longa L*. against Oxidative Stress-Induced Toxicity: Improvement Mechanisms of Liver Detoxification

**DOI:** 10.1155/2020/9535731

**Published:** 2020-04-19

**Authors:** Mohammed A. Hasan

**Affiliations:** Biology Science Department, College of Education for Girls, Thi-Qar University, Nasiriyah, Iraq

## Abstract

The rhizomes of *Curcuma longa L*. (CL) have been widely used in herbal medicines worldwide. It has been shown to possess prophylactic effects against oxidative stress. However, there is a paucity of information regarding the protective role of CL against oxidative stress in the absence of toxic agents. The aim of the study was to elucidate the antioxidative stress pharmacodynamics of CL. Eighteen 12-week-old Sprague-Dawley rats weighing about 300 ± 25 gm were divided equally into six groups. Four of the groups were supplemented with CL at 100 mg/kg b.w./day orally (P.O.) and labeled as 1^st^, 3^rd^, 5^th^, and 6^th^ day groups. The PCx (positive control) group was given distilled water orally, and the NCx (negative control) group rats were provided with food and water ad libitum. Blood samples were collected, and rats were sacrificed on days 1, 3, 5, and 6 (2 h) posttreatment. The blood was used for oxidative stress enzyme analysis (SOD, GSH-Px, and MDA) and liver (ALT) and kidney (creatinine) function assay, and the liver was dissected for histology. The results revealed that CL exhibited an antioxidative stress effect in the liver and kidneys as indicated by the low levels of ALT and creatinine. In response to antioxidant enzymes, especially that of the 3^rd^-day treatment group, an increase in SOD and GSH-Px indirectly caused an alleviation of oxidative stress, leading to a much lower level of MDA. It was concluded that treatment with CL at 100 mg/kg b.w./per day for three consecutive days demonstrated the highest efficacy in abating oxidative stress in rats.

## 1. Introduction

Conventional drugs and treatment have been associated with adverse side effects and other complications, such as drug resistance. Moreover, some of the existing conventional drugs are not sufficient or effective in providing a complete treatment of certain diseases. Therefore, this warrants further investigation into the identification and discovery of new drugs for alternative therapy, either to complement or replace existing conventional drugs [1, 2]. Thus, traditional medicines of plant origin have recently garnered more attention because of several factors such as easy availability, safety, affordability, and efficacy as well as cultural acceptability [3–5]. The medicinal properties, a potential mechanism of action, toxicological studies, and safety evaluation, of a lot of the plants in use today, remain unclear [6–8]. Among the different medicinal plants, *Curcuma longa L.* (CL) is also known as Zingiberaceae, turmeric, or yellow ginger, which has been used in alternative medicinal systems like the Unani, Ayurvedic, and Chinese medicine to treat a liver disorder, hypercholesterolemia, and GIT problems [9]. Aims of this study are to determine the potential protective effects of powdered dried rhizomes of CL on antioxidative status, particularly, SOD and GSH-Px, and lipid peroxidation as well as cytosolic liver enzymes, to evaluate the morphological, histopathological, and ultrastructural features in the liver associated with oral administration of CL, and to elucidate the mechanisms and protective pathways of CL in abating free radical-induced hepatotoxicity in rats.

## 2. Materials and Methods

### 2.1. Experimental Design

Animals were equally divided into six groups, four of them called the treatment groups and two control groups. The treatment groups consisted of (A) 1^st^, (B) 3^rd^, (C) 5^th^, and (D) 6^th^ day groups, which were supplemented with a CL aqueous suspension at a dose of 100 mg/kg/per day orally. Group E or the positive control groups were given distilled water orally, while Group F served as a negative control group. On days 1, 3, 5, and 6 posttreatment, animals were anaesthetized to the collection of blood samples, for biochemical assay studies, and subsequently sacrificed 2 h after each treatment.

### 2.2. Biochemical Analysis and Histological Examination

The plasma, serum, and hemolysate RBCs were extracted from blood samples, for spectrophotometric determination: plasma malondialdehyde (MDA) concentration as an oxidative stress marker [10, 11], estimation of erythrocyte-superoxide dismutase (E-SOD) activity as the first intracellular defense against free radicals [12], and estimation of erythrocyte-glutathione peroxidase (E-GSH-Px) activity as a master antioxidant enzyme [13].

Livers were resected and cleaned from surrounding adipose tissue. After washing gently with 0.9% physiological saline, and blotted dry, somatic index (SI) determination was carried out. During necropsy, the focus was on the liver. Pathological changes, if found, were scored from 0–3; {0 = no lesion (no significant alterations) and 3 = most severe}. The scoring was carried as follows: no significant lesions = 0 and 5–10% organs affected considered mild and scored (1) point, 11–25% considered moderate and scored (2) points, and over 25% considered severe and scored (3) points.

### 2.3. Statistical Analysis

Mean total clinical signs, biochemical tests, gross, histological lesions, and scores were summarized and subjected to the Mann–Whitney test. All statistical procedures were estimated using the predictive analysis software 20 (PASW version 22; SPSS Inc., Chicago, IL, USA) and tested at 5% level of significance.

## 3. Results

### 3.1. Clinical Signs, Body Weight, Feed Intake, and Water Consumption

No clinical abnormalities were observed in all of the rat's groups during the experimental period. There were no statistical differences in the treated groups compared to the negative control group, which was indicative of the healthy status of rats, following CL supplementation.

### 3.2. Biochemical Test Result

**Figure 1:**
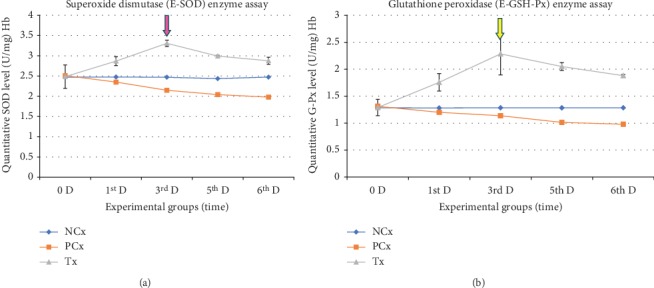
Effects of 100 mg/kg/day (P.O.) of CL, for six successive days, (left-line graph) E-SOD (U/mg Hb) level, and (right-line graph) E-G-Px (U/g Hb) level are plotted. The pink and yellow arrows indicate the point when the enzymes reached its peak (the 3^rd^ day after administration).

**Figure 2:**
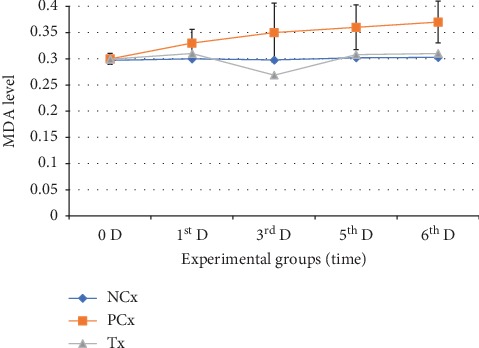
Effects of 100 mg/kg/day (P.O.) of ZO, for six successive days on the oxidative stress marker enzyme level (MDA). The mean ± SE of plasma-MDA (nmol/mL) levels is plotted. The green arrow indicates the point when enzyme reached the lowest level (the 3^rd^ day after administration).

Oral treatment of healthy rats with 100 mg/kg/day per os (P.O.) CL for 6 successive days induced a significant increase in erythrocytes (SOD and G-Px) and a significant decrease in plasma (MDA), during the third day only (group B) of administration when compared with the NCx group. While in the fifth and sixth days of administration, these changes gradually started to return to the normal values. The inhibitory effect of CL on plasma MDA enzyme was associated with an increase in erythrocyte (SOD and G-Px) (Figures 1 and 2).

It was observed that levels of serum enzymes (ALT and CREA) were affected by supplementation with 100 mg/kg/day (P.O.) CL. Furthermore, the results suggest that supplementation with CL does not induce hepatonephrotoxicity, especially in the cell membrane. In contrast, there was a slight increase in the level of these enzymes, as detected in PCx as compared to NCx.

## 4. Histopathological Findings

### 4.1. Gross Evaluations (Organ Weight, Size, Color, and Consistency)

According to the somatic index determinations (SI) for rats, there were no significant changes in liver weight observed in all experimental animals as compared to NCx. Also, following palpation and visual examination of these organs, they exhibited normal consistency, color, and size (no gross abnormalities related to 100 mg/kg CL fresh suspension administration was detected in the 6 days).

### 4.2. Microscopic Analysis

**Figure 3:**
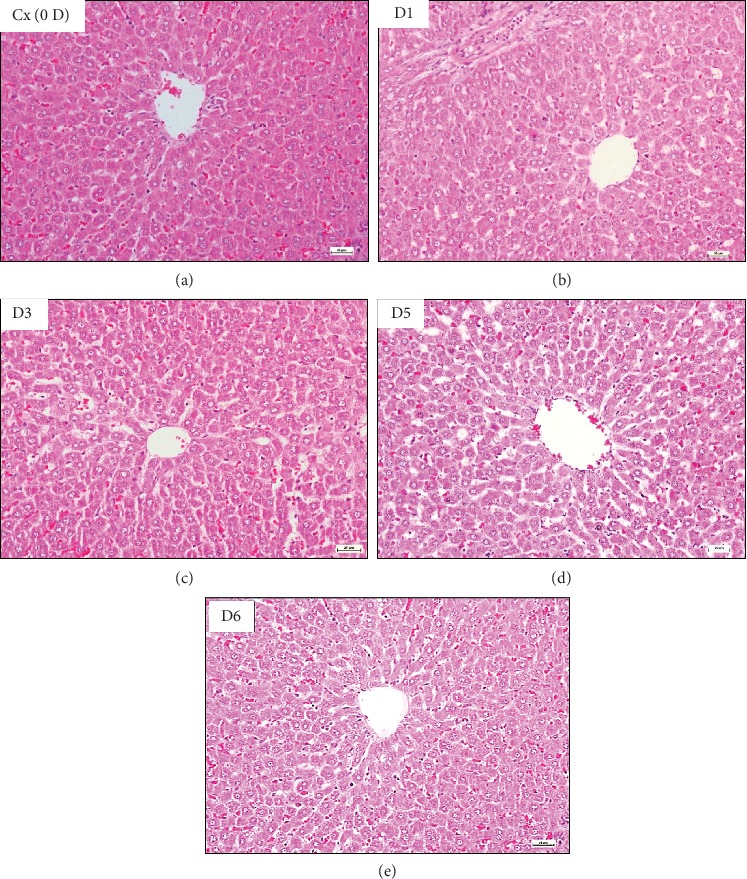
Photomicrograph of liver from control and CL (100 mg/kg) groups shows a normal limit, represented by normally arranged hepatocytes around the central vein together with normal sinusoidal capillaries (H&E, 20x).

**Figure 4:**
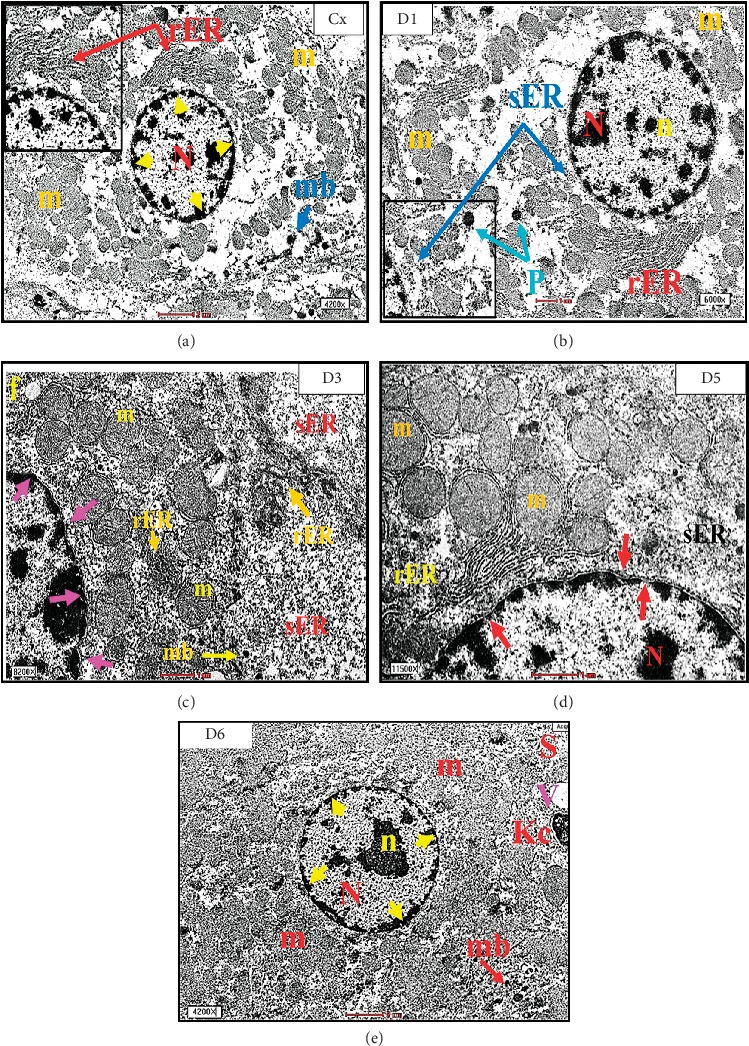
A transmission electron micrograph (TEM) of the rat liver showing normal appearance of hepatocyte with euchromatic nucleus (N) as well as developed nuclear membrane (yellow head arrow), and the cytoplasm contains normal mitochondria (m), an array of rough endoplasmic reticulum (rER) and smooth endoplasmic reticulum (sER), and normal peroxisome (P).

The results of the histopathologic examination (lesion scoring) are shown in Table 1. The histological architecture of all rats' groups revealed the normal limit of hepatic tissue with normal ultrastructural appearance of hepatocyte as euchromatic nucleus and developed a nuclear membrane, and the cytoplasm contains normal mitochondria and an array of rough endoplasmic reticulum (rER) and smooth endoplasmic reticulum (sER), with normal peroxisome (Figures 3 and 4).

### 4.3. Correlations among Histopathological Lesion Score and Biochemical Parameters Like Health Status and Liver Somatic Index

The correlation coefficients between and among all selected histopathological lesions at variable biochemical parameters, health status, and somatic index were very strong, and significant (*p* < 0.01) correlation existed between histopathological scoring and body weight (*r* *=* −0.903), feed intake (*r* *=* −0.975), water consumption (*r* *=* −0.928), MDA (*r* *=* 0.929), CREA (*r* *=* 0.912), and ALT (*r* *=* 0.974). Meanwhile, the strong and also significant (*p* < 0.01) correlation existed among histopathological scoring and liver weight (*r* *=* 0.895), SOD (*r* *=* −0.894), and G-Px (*p* < 0.05) (*r* *=* −0.770). Almost the value of “*r*” for each parameter was near to one that clearly showed how strong the relationship among these factors is.

## 5. Discussion

In the present study, the decreased activity of G-Px could be attributed to a reduction in reduced glutathione GSH (by oral gavage induced-oxidative stress), which could result in an increase in peroxides. A reduction in glutathione GSH leads to a proportional decrease in H_2_O_2_ detoxification, by glutathione peroxidase [14]. Therefore, the balance of this enzyme system is essential, in removing superoxide anion and peroxides generated in the liver. When animals are exposed to stress, they are more vulnerable and susceptible to infectious pathogens and subsequent shedding [15]. To the best of our knowledge, this study was the first to demonstrate the effects of oral administration of 100 mg/kg (b.w.)/per day of CL fresh suspension for six consecutive days, on Sprague-Dawley rats. The results revealed that treatment up to the 3^rd^ day was highly efficacious in increasing antioxidant enzymes and abating oxidative stress and lipid peroxidation in CL-treated groups (Txs). These findings are of utmost importance, as CL is currently being subjected to several pharmacological screening studies as a potential candidate for the prevention of carcinogenesis [16]. The treatment groups (Txs) of rats with 100 mg/kg CL induced an increase in antioxidant enzyme (E-SOD and E-G-Px) activity, which peaked on the 3^rd^ day of administration. The prophylactic role and antioxidant effects of CL were further supported by the significant reduction of lipid peroxidation and oxidative stress during the same period in Tx groups, indicated by the levels of plasma malondialdehyde (MDA). The inhibition of lipid peroxides as well as an increase in SOD and G-Px activity, among the treatment groups (Txs) immediately after oral administration of CL (which peaked after three days) in the current investigation, could presumably be explained as protective effects of CL against oxidative damage. Furthermore, a decrease observed in MDA levels in the Tx groups following CL supplementation is suggestive that CL has appreciable free-radical scavenger properties and possesses the ability to suppress lipid peroxidation as similarly reported in previous studies [17]. Regulation of lipid peroxidation, radical scavenging, and antioxidant status may be one of the important mechanisms by which CL exerts its toxic inhibitory effect. The results of this study corroborate with the results of previous studies in which the effects of aqueous extract of dried *C. longa* roots were investigated [18, 19]. The positive effects of *C. longa* such as inhibiting lipid peroxidation of biological membranes and activities of liver enzymes and contributing to the antioxidant defense system, which has been reported in the previous studies, could be attributed to the main active constituents of the turmeric “curcuminoids” [20]. However, from the fourth day onwards following CL administration for the Tx groups, a decline in SOD and G-Px levels to the normal value was observed. Although no studies have evaluated the effects of CL feeding following stress induced by oral gavage even in the absence of toxic agents, the decline observed could be associated with the consequence of less availability of free radicals or superoxide radicals (substrate for these antioxidant enzymes), which decreased gradually after administration of CL. It could be due to the reported potent superoxide radical-scavenging property of CL, which has been shown to be as effective as superoxide dismutase. The findings of this trial are consistent with a previous study that demonstrated how prior treatment with CL roots resulted in a reduction in the levels of lipid peroxidation and oxidative stress [21]. Other studies reported similar results, in which oral administration effectively induced an increase in quinone reductase and glutathione transferase activity, even in the absence of toxic agents. They also reported that administration with CL inhibited ROS generation and caused an increase in the levels of antioxidant enzymes such as SOD and GSH [22]. The present study investigated the effect of CL on ALT and creatinine (CREA) as well as the pathological changes in the liver and kidneys (macro- and microscopic pathological changes). Supplementation of 100 mg/kg CL for six days did not induce significant changes in the biochemical parameters of liver and kidney function, and gross and histopathological examinations revealed the normal architecture of the liver. It was further supported by a decrease (no significant reduction) of serum ALT and creatinine level in Tx compared to NCx. While in the PCx group, there were slight gradual increases in the level of these enzymes from the first day of the experiment. These observed results could be due to the oral method of administration, which inherently could be the cause of adverse effects, such as increased oxidative stress. This induced toxicity even in the absence of toxic compounds, and due to the short duration of the experiment, histological changes were not observed. However, a decrease in the levels of these enzymes observed in the treatment groups, following administration of CL, was due to the antioxidative stress effect and free radical scavenger role of CL. The lesion score technique was employed, to confirm the findings, and the absence of pathological changes in the tissues, especially in the histological evaluation and lesion scores, confirmed the results. Previous studies also reported similar findings that CL rhizome extract increased the activity of SOD, GPx, and CAT enzymes [23]. Also, oral administration of aqueous extract of CL rhizomes did not cause significant changes in liver function, demonstrated by hepatic enzymes levels and histopathological changes in liver tissue [21, 23].

The current study showed that six days of 100 mg/kg CL (P.O.) administration did not cause rise to any toxic effects on vital organs, specifically the liver. However, prior feeding of CL induced protective effects against oxidative effects caused by oral gavage. Similar to our results, Zhang et al. [22, 24] also suggested that CL treatment increased the antioxidant defense system activity in experimentally induced diabetic rabbits. However, Amalraj et al. [20, 21, 25] concluded that CL caused a decrease in lipid peroxidation and an increase in antioxidant defense system activity in the CCl_4_-treated rats. Our findings are in accordance with previous reports of other related studies [17, 18, 23] that have suggested the protective role of CL powder, possibly due to the antioxidative effect of polyphenols and flavonoids present in the roots, that act as strong superoxide radicals and singlet oxygen quenchers. Also, we agree with Shakeri et al. [26] who concluded treatment with CL rhizome extract caused an elevation of GSH levels, which protects the membranes against oxidative damage through regulation of the redox status of protein in the membrane. Treatment with CL rhizomes even in the absence of toxic agents may enhance/reinforce antioxidant defense status of the body such as intracellular glutathione to induce antioxidative stress and free radical-scavenger activity, following CL administration. This could explain the reason why an increase and decrease in SOD and G-Px levels were observed in a very short duration (peaked at the 3^rd^ day of administration) and decreased to normal levels immediately after this period. This observation is in accordance with previous studies by Shakeri et al. [26] where antioxidant and free-radical scavenging activities of glutathione (GSH) and polyphenols and flavonoids present in the roots were observed, which resulted from the reaction that occurred between them and NADH and NADPH, following CL administration. The possibility of an intracellular nonenzymatic metabolic activation of curcuminoids dependent on GSH, NADH, or NADPH has also been suggested that could act as a “cellular switch,” which could regulate cellular antioxidant defenses. The results of this study are in agreement with the observations of other related studies [27, 28], which reported curcuminoids induction of the expression and/or activity of GST, GSH-Px, SOD, and glutathione reductase. Consequently, CL improved plasma and liver antioxidant ability and increased expression of liver antioxidant genes.

## 6. Conclusion

These results provided evidence that CL rhizomes are a promising medicinal plant with many prophylactic and therapeutic properties, and it appeared to be safe with possible protective properties against free radical-induced hepatonephropathy. When administered orally, it led to reduced impairment of liver functions by the preservation of intracellular GSH-Px, which could explain the hepatoprotective effect, decreased MDA concentration, and increased SOD and GSH-Px concentrations, as a result of its antioxidant nature. It also prevented lipid peroxidation-induced liver damage, by its radical scavenging nature. With the evidence of normal histological findings for all treated groups, it suggests that CL does not have any toxic effects on the liver and kidneys at a dose 100 mg/kg/day/orally for six consecutive days.

## Figures and Tables

**Table 1:** Lesion scoring for tissues after CL supplementation for six consecutive days.

Organ	Lesion	Control		1^st^ day	3^rd^ day	5^th^ day	6^th^ day
		NCx	PCx				
Liver	Congestion	0.00	0.00	0.00	0.03 ± 0.01	0.04 ± 0.01	0.05 ± 0.006
	Dilation of sinusoid	0.00	0.00	0.00	0.01 ± 0.006	0.01 ± 0.006	0.02 ± 0.01
	Inflammatory cell infiltration	0.00	0.00	0.00	0.00	0.01 ± 0.006	0.01 ± 0.013
	Hepatocellular degeneration	0.00	0.00	0.00	0.00	0.00	0.00
	Hepatocellular necrosis	0.00	0.00	0.00	0.00	0.00	0.00

Kidney	Congestion	0.00	0.00	0.00	0.00	0.01 ± 0.006	0.02 ± 0.011
	Interstitial leukocytic infiltration	0.00	0.00	0.00	0.00	0.00	0.00

Spleen	Congestion	0.00	0.00	0.00	0.00	0.01 ± 0.006	0.01 ± 0.006
	Lymphoid depletion SWP	0.00	0.00	0.00	0.00	0.00	0.00
	Mononuclear infiltration SRP	0.00	0.00	0.00	0.00	0.00	0.00
	Total lesion score	0.00	0.00	0.00	0.04	0.08	0.11

## Data Availability

The data used to support the findings of this study are included within the article.
